# The Pathogenic Potential of a Microbe

**DOI:** 10.1128/mSphere.00015-17

**Published:** 2017-02-22

**Authors:** Arturo Casadevall

**Affiliations:** Department of Molecular Microbiology and Immunology, Johns Hopkins Bloomberg School of Public Health, Baltimore, Maryland, USA; Duke University Medical Center

**Keywords:** formula, pathogenesis, virulence

## Abstract

Virulence is a microbial property that is realized only in susceptible hosts. There is no absolute measurement for virulence, and consequently it is always measured relative to a standard, usually another microbe or host. This article introduces the concept of pathogenic potential, which provides a new approach to measuring the capacity of microbes for virulence. The pathogenic potential is proportional to the fraction of individuals who become symptomatic after infection with a defined inoculum and can include such attributes as mortality, communicability, and the time from infection to disease. The calculation of the pathogenic potential has significant advantages over the use of the lethal dose that kills 50% of infected individuals (LD_50_) and allows direct comparisons between individual microbes. An analysis of the pathogenic potential of several microbes for mice reveals a continuum, which in turn supports the view that there is no dividing line between pathogenic and nonpathogenic microbes.

## Opinion/Hypothesis

The germ theory of disease posited that certain microbes caused specific diseases (e.g.,* Mycobacterium tuberculosis* caused tuberculosis). Furthermore, it was rapidly apparent that there were differences in virulence within a microbial species (1). Virulence is the relative capacity of a microbe to cause damage, with the term “relative” being a necessary component of the definition since there are no absolute measures of virulence (2). Virulence is always measured relative to a standard, such as another microbe or host. Today, most comparisons of virulence are done within a microbial species, with investigators comparing the virulence of the wild type to that of an experimentally modified strain, such as one with a gene deletion or mutation. Virulence is expressed only in a susceptible host, and its measurement is critically dependent on the host system used (3).

Historically, the measurement of virulence has relied on some quantifiable outcome on the host, such as mortality. The most commonly used measurement of virulence is the lethal dose required to kill 50% of infected hosts, referred to as the LD_50_. The LD_50_ measurement has the advantage that it allows comparisons across microbes, and the use of host death provides a nonequivocal endpoint. However, the LD_50_ has the considerable limitation that it is a coarse measure that is not applicable to those host-microbe interactions where host death does not occur or is a very rare outcome. Another limitation of the LD_50_ is that it is focused on mortality and thus misses other outcomes of infection, such as chronicity and latency. However, perhaps the greatest limitation of LD_50_ studies is lack of discriminatory power such that 10-fold differences in the size of the inoculum can lead to the same LD_50_ value (for an example, see reference 4).

Given the limitations of the LD_50_, some investigators have developed approaches for measuring virulence that are not dependent on mortality. Beynen et al. developed a symptomatic assessment scale that relied on measuring a variety of parameters in mice with gallstones (5). Soothill et al. used hypothermia as a reliable early indicator of mortality in mice and developed the HID_50_, or 50% hypothermia-inducing dose (6). These formulations, like the LD_50_, are useful for the specific situations for which they were developed but lack global applicability for comparison across microbes and potential hosts.

## 

### The pathogenic potential of a microbe.

The goal of this effort was to find a mathematical relationship that allows a quantitative measure of the capacity of a certain microbe for pathogenicity. To accomplish this, I propose the concept of the pathogenic potential (PP). In this phraseology, the word “pathogenic” retains the meaning proposed earlier (2) as the capacity of a microbe to cause damage in a host and the word “potential” acknowledges that no microbe can be pathogenic in the absence of a host. In considering the factors that contributed to the pathogenic potential, it was important to account for the fact that the damage incurred by a host during a host-microbe interaction was variable. Clinical symptoms manifest themselves only when the host-microbe interaction results in sufficient host damage to disrupt homeostasis, and death ensues when the damage is so overwhelming that it precludes repair and a return to homeostasis.

The pathogenic potential (PP) of a microbe is proposed to be proportional to the ratio of the fraction symptomatic (Fs) and the infecting inoculum (*I*): PP α Fs/*I*.

Since symptomatology can range from a passing malaise to death, this formulation does not capture the severe impact of host-microbe interactions associated with host mortality. To include host-microbe interactions that result in death, the ratio is modified by multiplying it by the 10th power of the mortality fraction: PP = (Fs/*I*)(10^*M*^).

In this formulation, the Fs/*I* term includes the microbial pathogenicity and host susceptibility factors essential for microbial pathogenesis, while the 10^*M*^ factor is an amplifier to account for host mortality, an extreme outcome of the host-microbe interaction. The Fs/*I* term is identical to that used previously in developing a formula for the weapon potential of a microbe (7). As will be illustrated below, when actual calculations are done, the *I* parameter to be used should be the smallest that produces symptoms/death, since progressively larger inocula associated with symptoms/death for all animals will result in a smaller PP. For the mortality amplification, the 10th power was used to allow for the possibility of host-microbe interactions with no mortality, in which case *M* = 0.0, which reduces 10^*M*^ to 1.0. For host-microbe interactions with 100% mortality, *M* = 1.0, which contributes 10. For example, a microbe that caused symptoms in 100% (Fs = 1.0) of those infected with one microbe (*I* = 1.0) and killed every infected host (*M* = 1.0) would have a PP of 10. On the other hand, for host-microbe interactions where there are no symptoms (Fs = 0.0), which de facto implies *M* = 0.0, the PP reduces to 0.0. For host-microbe interactions that produce no symptoms but result in lesions to the host, such as granulomas, one can replace the Fs for the fraction of individuals affected. The proposed PP essentially reduces to the inverse of the infecting inoculum when comparing infections with microbes when all hosts become symptomatic, consistent with the definition of virulence as the inverse of inoculum, which was used in the early days of the germ theory (8).

### Pathogenic potential and communicability (or transmissibility).

The PP formulation proposed above does not include the element of communicability. The reason for this omission was to develop a formulation that could be used to compare the pathogenic potentials of microbes independently of their communicability potential and also allow for the comparison of the effects of microbes on individual hosts independently of their effects on the entire population of susceptible hosts. The relationship between communicability and virulence can very complex (9), and it is relevant for some pathogenic microbes and not others. For *M. tuberculosis*, the capacity for communicability is dependent on its ability to cause lung damage and consequent dissemination through aerosolization of infective bacteria. However, the virulence of *Bacillus anthracis* is not dependent on communicability, and anthrax is not a transmissible disease. For other microbes, such as BK virus, communicability is common, but disease is extremely rare, unless the host is immune suppressed. Hence, the relationship between pathogenic potential and communicability is microbe specific, with virulence and communicability being dependent variables for some host-microbe relationships and not others.

The element of communicability is also relevant to have an impact on the host species, where it functions as an amplifier of threat. Given that communicability (*C*) is an amplifier, it can be added to the pathogenic potential as a multiplier term. Hence, a formulation for PP that includes the contribution of transmissibility (PP_*C*_) can be PP_*C*_ = (Fs/*I*)(10^*M*^)*C.*

Since there are no absolute values for *C*, it can be assigned a value ranging from 1 (not communicable) to 100 (every case leads to one or more new cases) based on epidemiological data. Separating PP from *C* allows these properties to be considered separately when comparing the outcome of host-microbe interactions in individuals and populations.

### The element of time.

Neither PP nor PP_*C*_ includes time to disease. The element of time is often a central determinant of how the pathogenic potential of a microbial species is perceived. When the words “fulminant” and “aggressive” are used in the context of infectious diseases, they usually connote an element of rapidity or shortness of time between infection and disease. Cryptococcosis, HIV infection, and disseminated anthrax have in common close to 100% mortality in affected individuals if untreated, but these diseases are often viewed differently with respect to their relative virulence because the times to death differ greatly. Diseases that kill a host in a short time after infection create a much greater impression than those where there is a long time interval between infection and demise. For example, anyone who has witnessed a case of meningococcal meningitis and/or sepsis cannot but be impressed with how fulminant that disease can be, which can progress from a mild condition to death in a matter of hours. For those who want to consider the element of time, this can be easily included in either the PP or PP_*C*_ formulations by multiplying these calculated parameters by 1/*T*, where *T* is time. Consequently, infectious diseases where the times to symptoms are shorter will yield a higher PP or PP_*C*_ than those that take a long time from infection to disease.

### The exceptionality of certain toxin-mediated diseases.

Toxin-mediated diseases are different from other infectious diseases in that they can trigger symptoms irrespective of the immune system function (2), and for some diseases, the connection between microbe and host can be both temporally and/or spatially separate. Furthermore, these are a diverse set of diseases for which the PP is applicable to some but not others. For example, the proposed PP formulation works for tetanus, where skin infection with an inoculum of *Clostridium tetani* results in certain fraction of infected individuals developing tetanus. In contrast, for botulism and staphylococcal food poisoning, the disease results from the ingestion of a preformed toxin whose production is temporally and spatially separate from introduction into the host, and the PP formulation proposed above is not applicable, since there is no relevant inoculum. However, the PP formulation can be modified to substitute the inoculum for the toxin dose ingested to yield PP_*D*_. Hence, for toxin-mediated diseases where the toxin in produced within the host as a consequence of infection, the PP applies, while PP_*D*_ can be used for microbial diseases resulting from the ingestion or inoculation of preformed toxin. Like PP, the PP_*D*_ can be modified to take into account the element of time but when these diseases are not communicable and *C* can be assigned the value of 1.0.

### Calculations using the pathogenic potential formulation.

Using the PP formulation with experimental data demonstrates advantages as well as some caveats in its application. The first example will use published data on the virulence of *Salmonella enterica* serovar Typhimurium strains differing in lipopolysaccharide side chains (4). Table 1 lists the original data found in Table 2 of reference 4 and the calculated PP. One immediate advantage is that the PP calculation remains useful for making comparisons between strains when there is difference between the assumed inocula and the actual inocula as revealed by postinfection plating or plaque assays. Another advantage is that the PP calculation discriminates between conditions where 10-fold differences in inoculum yielded the same LD_50_. For example, for strain 2201, there is 50% mortality with bacterial inocula of both 10^5^ and 10^4^ producing identical LD_50_ values, but the PP shows a 10-fold difference between these conditions because it includes the inoculum in the denominator. Furthermore, the calculation of PP allows much finer discrimination for virulence differences than is apparent in simple inoculum versus mortality comparisons. In this regard, it is clear that when comparing strains, the PP can differ depending on the inoculum. For example, strain 2201 appears to be most virulent at the lowest inoculum that causes any mortality, but comparison of the calculated PP of this strain and that of strain 2203 reveals the latter to have a greater PP with the larger inocula. On the other hand, the inclusion of the Fs term in the PP formulation results in major caveats that must be taken into account to avoid calculation foibles. In calculating the PP for mice, the first issue encountered is that in a mortality study, the Fs is not known, and even if the experimenters had set out to measure this parameter, it is difficult to detect minor symptoms in mice. Hence, for inocula where some mice died, in the sample calculations shown in Table 1 the Fs was set at 1.0 based on the assumption that if the experimental infection was sufficient to kill some mice, then there was a high likelihood that some symptoms were experienced by all survivors. However, setting Fs = 1 clearly does not work when the inoculum is too low to result in any deaths since that would have the effect of producing a very high PP even if all the mice were only transiently ill. Hence, when using the PP formulation, one must be to be careful to consider the type of symptoms measured and to use judgment on the input values.

**TABLE 1:** Analysis of pathogenic potential of *Salmonella enterica* serovar Typhimurium in mice based on mortality data^a^

Strain	Result for inoculum of^b^:									
	5 × 10^7^		5 × 10^6^		5 × 10^5^		5 × 10^4^		5 × 10^3^	
	*M*	PP	*M*	PP	*M*	PP	*M*	PP	*M*	PP
2201^c^	1.0	1.7 × 10^−7^	1.0	1.7 × 10^−6^	0.5	5.3 × 10^−6^	0.5	5.3 × 10^−5^	0.0	0
2202	1.0	2.0 × 10^−7^	0.8	1.3 × 10^−6^	0.6	8.0 × 10^−6^	0.1	2.5 × 10^−5^	0.0	0
2203^d^	1.0	2.5 × 10^−7^	1.0	2.5 × 10^−6^	0.9	2.0 × 10^−5^	0.2	4.0 × 10^−5^	0.0	0
2204	0.8	1.3 × 10^−7^	0.7	1.0 × 10^−6^	0.1	2.5 × 10^−6^	0.0	0	0.0	0
2205	0.8	1.3 × 10^−7^	0.5	6.3 × 10^−7^	0.2	3.2 × 10^−6^	0.2	3.2 × 10^−5^	0.0	0
2206	1.0	2.0 × 10^−7^	0.9	1.6 × 10^−6^	0.2	3.2 × 10^−6^	0.0	0	0.0	0

a The original data were published in Table 2 in reference 4. There was no specific reason for selecting this study apart from that it included sufficient data to make the point that PP can discriminate in situations where the LD_50_ does not discriminate, as is evident for strain 2201, where 50% mortality was obtained with inocula that differed by 10-fold.

b *M* stands for mortality, and the numbers given are the fractional mortality data for 10 mice. In calculating the PP, the Fs was assumed to be 1.0 under all conditions where there was at least one death and 0.0 when no death was observed.

c The infective inocula for this strain were subsequently found to be 6 × 10^7^, 6 × 10^6^, etc.

d The infective inocula for this strain were subsequently found to be 4 × 10^7^, 4 × 10^6^, etc.

### Implications for microbial pathogenesis.

The PPs of several microbes for mice were calculated using information from the literature (Table 2). The listing of the PP for these microbes reveals a continuum in the ability of microbes to disease in mice (Table 2), and it can vary depending on mouse genetics (Table 3). This in turn argues against a fundamental qualitative difference between so-called “pathogenic” and “nonpathogenic” microbes, since one can always increase the inoculum to create conditions under which some fraction of infected hosts are symptomatic. Thus, saprophytic microbes and those that exist in a commensal state in an immunocompetent host can cause disease if the inoculum is high enough. This was demonstrated in a famous self-experiment when an investigator ingested 10^12^ CFU of *Candida albicans* and developed a transient illness with fever, shivering, and severe headache that was accompanied by the presence of yeast in blood and urine, indicating rapid dissemination from the intestine (10). Consequently, classifications of microbes into pathogenic and nonpathogenic sets can be futile exercises since such categorizations apply only for defined inocula in certain hosts. On the other hand, microbes can be stratified according to their pathogenic potential, provided that information is available on the Fs per a given inoculum. Incidentally, the PP formulation is applicable in those situations where the microbe in question changes its relationship with a host from commensal to disease, as a result of a change in the host. Continuing with the example of *C. albicans*, this microbe exists in a commensal state in most human hosts, but when these hosts are given antimicrobial drugs that affect the indigenous microbiota, the fungal burden can increase tremendously and can lead to disseminated candidiasis. The *C. albicans* burden in the gut of individuals treated with antimicrobial drugs can reach 10^9^/ml, a number that approaches the ingestion dose of Krause when the entire gut volume is taken into account (11).

**TABLE 2:** Pathogenic potential calculations for several microbes in mice^a^

Microbe or condition	Mortality	Inoculum	PP	Reference
Theoretical maximum	1.0	1.0 × 10^0^	1.0 × 10^1^	This work
*Francisella tularensis*	0.5	2.0 × 10^0^	1.6 × 10^0^	13
*Bacillus anthracis*	0.5	2.6 × 10^0^	1.2 × 10^0^	14
*Brucella suis*	0.5	3.8 × 10^0^	8.3 × 10^−1^	15
*Toxoplasma gondii*	0.5	1.5 × 10^1^	2.1 × 10^−1^	16
*Coccidioides immitis*	0.5	1.7 × 10^1^	1.9 × 10^−1^	17
*Klebsiella pneumoniae*	0.5	1.9 × 10^1^	1.7 × 10^−1^	18
*Streptococcus pneumoniae*	0.5	3.0 × 10^1^	1.1 × 10^−1^	19
*Yersinia pestis*	0.5	3.7 × 10^1^	8.5 × 10^−2^	20
*Cryptococcus neoformans*	0.5	5.1 × 10^1^	6.2 × 10^−2^	21
*Vibrio vulnificus*	0.5	7.5 × 10^1^	4.2 × 10^−2^	22
Herpes simplex virus	0.5	2.2 × 10^2^	1.4 × 10^−2^	23
*Escherichia coli*	0.5	1.0 × 10^3^	3.2 × 10^−3^	24
*Candida albicans*	0.5	6.6 × 10^3^	4.8 × 10^−4^	25
Murine cytomegalovirus	0.5	5.0 × 10^4^	6.3 × 10^−5^	23
*Aspergillus fumigatus*	0.25	6.0 × 10^4^	3.0 × 10^−5^	26
Group B streptococcus	0.5	6.3 × 10^4^	5.0 × 10^−5^	27
Murine adenovirus	0.5	1.0 × 10^5^	3.2 × 10^−5^	28
*Listeria monocytogenes*	0.5	2.4 × 10^5^	1.3 × 10^−5^	29
*Nocardia asteroides*	0.5	8.5 × 10^5^	3.7 × 10^−6^	30
*Shigella sonnei*	0.5	1.6 × 10^6^	2.0 × 10^−6^	31
*Naegleria fowleri*	0.75	5.0 × 10^6^	1.1 × 10^−6^	32
*Bacillus cereus*	0.5	1.0 × 10^7^	3.2 × 10^−7^	14
*Staphylococcus saprophyticus*	0.5	2.7 × 10^7^	1.2 × 10^−7^	33
*Bacillus thuringiensis*	0.5	1.1 × 10^7^	2.9 × 10^−7^	14
*Pseudomonas aeruginosa*	0.5	5.0 × 10^7^	6.3 × 10^−8^	34
*Legionella pneumophila*	0.5	6.7 × 10^7^	4.7 × 10^−8^	35
*Staphylococcus epidermidis*	0.5	6.0 × 10^7^	5.3 × 10^−8^	33
*Staphylococcus aureus*	0.5	1.0 × 10^8^	3.2 × 10^−8^	36
*Haemophilus influenzae* type B +	0.5	2.0 × 10^8^	1.6 × 10^−8^	37
mucin^b^	0.5	3.4 × 10^4^	9.3 × 10^−5^	37
*Enterococcus faecalis*	0.5	2.6 × 10^8^	1.2 × 10^−8^	38

a The PP calculation used literature information on the mortality of mice infected with these microbes. For the calculation, Fs was assumed to 1.0 in all instances, which means that the PP reduces to the inverse of the inoculum modified by the mortality parameter. In those studies where there were multiple microbial strains and different mouse strains, the values used are those that had the largest effects. The PP calculations in this table are for illustrative purposes only, and the reader is cautioned that the studies listed above used different mouse strains and infection routes. Hence, comparisons among the listed microbes should be done cautiously, and the major goal of this listing is to show that these lie on a continuum with regard to their pathogenicity for mice.

b Mixing the inoculum with mucin reduces the dose needed to cause mortality as the mucin presumably interferes with host defense mechanisms.

**TABLE 3:** The pathogenic potential of a microbe varies with the genetic background of the mouse strain^a^

Microbe	Mouse strain	Mortality	Inoculum	PP	Reference
*L. monocytogenes*	C57BL/6	0.5	9.0 × 10^5^	3.5 × 10^−6^	39
	B10.D2	0.5	2.2 × 10^5^	1.4 × 10^−5^	39
	B10.A	0.5	2.2 × 10^5^	1.4 × 10^−5^	39
	BALB/c	0.5	3.9 × 10^3^	8.1 × 10^−3^	39
	CBA	0.5	5.0 × 10^3^	6.3 × 10^−4^	39
	A/WySn	0.5	8.0 × 10^3^	4.0 × 10^−4^	39
	(C57BL/6 × BALB/c)F1	0.5	3.4 × 10^4^	9.3 × 10^−5^	39
*B. anthracis*	A/J	0.5	2.6 × 10^0^	1.2 × 10^0^	40
	C3H/HeJ	0.5	5.6 × 10^0^	5.6 × 10^−1^	40
	BALB/cJ	0.5	6.6 × 10^0^	4.8 × 10^−1^	40
	C58J	0.5	9.0 × 10^0^	3.5 × 10^−1^	40
	C57BL/6J	0.5	1.4 × 10^1^	2.3 × 10^−1^	40
	C57L/J	0.5	2.2 × 10^1^	1.4 × 10^−1^	40
Sendai virus	129/ReJ 1	0.5	3.2 × 10^0^	9.9 × 10^−1^	41
	SWR/J	0.5	5.0 × 10^2^	6.3 × 10^−3^	41
	C58/J	0.5	1.5 × 10^3^	2.1 × 10^−3^	41
	C57BL/6J	0.5	2.5 × 10^4^	1.3 × 10^−4^	41
	SJL/J	0.5	1.0 × 10^5^	3.2 × 10^−5^	41
*C. immitis*	BALB/cAnN	0.5	4.6 × 10^1^	6.9 × 10^−2^	17
	C57BL/10N	0.5	5.9 × 10^2^	5.4 × 10^−3^	17
	C57BL/6N	0.5	6.8 × 10^2^	4.7 × 10^−3^	17
	DBA/2NX1	0.5	1.8 × 10^5^	1.8 × 10^−5^	17

a The PP calculation used literature information for the mortality of mice infected with these microbes. For the PP calculation, Fs was assumed to 1.0 in all instances, which means that the PP reduces to the inverse of the inoculum modified by the mortality parameter. The PP calculations in this table are for illustrative purposes only, and the reader is cautioned that the studies listed above used different infection conditions. Hence, comparisons among the listed microbes should be done cautiously, and the major goal of the listing in this table is to show that how the changing the host can change the PP.

In summary, the proposed formulation for the PP of a microbe takes into account the contributions of both the host and the microbe to microbial pathogenesis. The infective inoculum is a function of both the intrinsic virulence of a microbe and host susceptibility. Microorganisms endowed with the capacity to disrupt host defenses (virulence factors) tend to require smaller inocula to cause disease in nonimmune hosts. PP provides a new means to rank microbes with regard to their relative virulence. The continuity in PP implies the absence of a clear defining line between so-called “pathogens” and “nonpathogens,” which highlights the futility of asking questions, such as “What is a pathogen?” (12) In that light, the PP calculation reinforces the suggestion to focus on the outcome of the host-microbe interaction rather than the participants (12).

## References

[1] Zinsser H 1914 Infection and resistance, p 1–27. Macmillan Company, New York, NY.

[2] Casadevall A, Pirofski LA 1999 Host-pathogen interactions: redefining the basic concepts of virulence and pathogenicity. Infect Immun 67:3703–3713. 10417127 10.1128/iai.67.8.3703-3713.1999 PMC96643

[3] Casadevall A, Pirofski L 2001 Host-pathogen interactions: the attributes of virulence. J Infect Dis 184:337–344. doi:10.1086/322044. 11443560

[4] Valtonen VV 1970 Mouse virulence of Salmonella strains: the effect of different smooth-type O side-chains. J Gen Microbiol 64:255–268. doi:10.1099/00221287-64-3-255. 4930444

[5] Beynen AC, Baumans V, Bertens AP, Havenaar R, Hesp AP, Van Zutphen LF 1987 Assessment of discomfort in gallstone-bearing mice: a practical example of the problems encountered in an attempt to recognize discomfort in laboratory animals. Lab Anim 21:35–42. 3560862 10.1258/002367787780740770

[6] Soothill JS, Morton DB, Ahmad A 1992 The HID50 (hypothermia-inducing dose 50): an alternative to the LD50 for measurement of bacterial virulence. Int J Exp Pathol 73:95–98. 1576081 PMC2002468

[7] Casadevall A, Pirofski LA 2004 The weapon potential of a microbe. Trends Microbiol 12:259–263. doi:10.1016/j.tim.2004.04.007. 15165603 PMC7133335

[8] Zinsser H, Bayne-Jones S 1939 A textbook of bacteriology, 8th ed. D. Appleton-Century Company Inc, New York, NY.

[9] Lipsitch M, Moxon ER 1997 Virulence and transmissibility of pathogens: what is the relationship? Trends Microbiol 5:31–37. doi:10.1016/S0966-842X(97)81772-6. 9025233

[10] Krause W, Matheis H, Wulf K 1969 Fungaemia and funguria after oral administration of Candida albicans. Lancet i:598–599. 10.1016/s0140-6736(69)91534-7 4180129

[11] Krause RM, Dimmock NJ, Morens DM 1997 Summary of antibody workshop: the role of humoral immunity in the treatment and prevention of emerging and extant infectious diseases. J Infect Dis 176:549–559. 9291299 10.1086/514074

[12] Pirofski LA, Casadevall A 2012 Q and A: what is a pathogen? A question that begs the point. BMC Biol 10:6. doi:10.1186/1741-7007-10-6. 22293325 PMC3269390

[13] Fortier AH, Slayter MV, Ziemba R, Meltzer MS, Nacy CA 1991 Live vaccine strain of Francisella tularensis: infection and immunity in mice. Infect Immun 59:2922–2928. 1879918 10.1128/iai.59.9.2922-2928.1991 PMC258114

[14] Lamanna C, Jones L 1963 Lethality for mice of vegetative and spore forms of Bacillus cereus and Bacillus cereus-like insect pathogens injected intraperitoneally and subcutaneously. J Bacteriol 85:532–535. 14042929 10.1128/jb.85.3.532-535.1963 PMC278179

[15] Zhao WR, Wendoso, Hasi, Qin YX, Weng W, Lu SL 1989 Selection of a Brucella vaccine strain of low residual virulence by chemical mutagenesis. J Med Microbiol 30:143–148. doi:10.1099/00222615-30-2-143. 2795638

[16] Lee YH, Kasper LH 2004 Immune responses of different mouse strains after challenge with equivalent lethal doses of Toxoplasma gondii. Parasite 11:89–97. 15071833 10.1051/parasite/200411189

[17] Kirkland TN, Fierer J 1983 Inbred mouse strains differ in resistance to lethal Coccidioides immitis infection. Infect Immun 40:912–916. 6852925 10.1128/iai.40.3.912-916.1983 PMC348138

[18] Berendt RF 1978 Relationship of method of administration to respiratory virulence of Klebsiella pneumoniae for mice and squirrel monkeys. Infect Immun 20:581–583. 97232 10.1128/iai.20.2.581-583.1978 PMC421896

[19] Yother J, Forman C, Gray BM, Briles DE 1982 Protection of mice from infection with *Streptococcus pneumoniae* by anti-phosphocholine antibody. Infect Immun 36:184–188. 7076292 10.1128/iai.36.1.184-188.1982 PMC351201

[20] Anderson GW Jr, Leary SE, Williamson ED, Titball RW, Welkos SL, Worsham PL, Friedlander AM 1996 Recombinant V antigen protects mice against pneumonic and bubonic plague caused by F1-capsule-positive and -negative strains of Yersinia pestis. Infect Immun 64:4580–4585. 8890210 10.1128/iai.64.11.4580-4585.1996 PMC174416

[21] Clancy CJ, Nguyen MH, Alandoerffer R, Cheng S, Iczkowski K, Richardson M, Graybill JR 2006 Cryptococcus neoformans var. grubii isolates recovered from persons with AIDS demonstrate a wide range of virulence during murine meningoencephalitis that correlates with the expression of certain virulence factors. Microbiology 152:2247–2255. doi:10.1099/mic.0.28798-0. 16849791

[22] Oliver JD, Bockian R 1995 In vivo resuscitation, and virulence towards mice, of viable but nonculturable cells of Vibrio vulnificus. Appl Environ Microbiol 61:2620–2623. 7618873 10.1128/aem.61.7.2620-2623.1995 PMC167533

[23] Shellam GR, Flexman JP 1986 Genetically determined resistance to murine cytomegalovirus and herpes simplex virus in newborn mice. J Virol 58:152–156. 3005643 10.1128/jvi.58.1.152-156.1986 PMC252887

[24] Rowley D 1954 The virulence of strains of Bacterium coli for mice. Br J Exp Pathol 35:528–538. 13230409 PMC2073678

[25] Wingard JR, Dick JD, Merz WG, Sandford GR, Saral R, Burns WH 1982 Differences in virulence of clinical isolates of Candida tropicalis and Candida albicans in mice. Infect Immun 37:833–836. 6749689 10.1128/iai.37.2.833-836.1982 PMC347607

[26] Smith GR 1972 Experimental aspergillosis in mice: aspects of resistance. J Hyg 70:741–754. 4567315 10.1017/s0022172400022580 PMC2130273

[27] Wessels MR, Butko P, Ma M, Warren HB, Lage AL, Carroll MC 1995 Studies of group B streptococcal infection in mice deficient in complement component C3 or C4 demonstrate an essential role for complement in both innate and acquired immunity. Proc Natl Acad Sci U S A 92:11490–11494. 8524789 10.1073/pnas.92.25.11490 PMC40427

[28] Guida JD, Fejer G, Pirofski LA, Brosnan CF, Horwitz MS 1995 Mouse adenovirus type 1 causes a fatal hemorrhagic encephalomyelitis in adult C57BL/6 but not BALB/c mice. J Virol 69:7674–7681. 7494276 10.1128/jvi.69.12.7674-7681.1995 PMC189708

[29] Pine L, Malcolm GB, Plikaytis BD 1990 Listeria monocytogenes intragastric and intraperitoneal approximate 50% lethal doses for mice are comparable, but death occurs earlier by intragastric feeding. Infect Immun 58:2940–2945. 2117576 10.1128/iai.58.9.2940-2945.1990 PMC313591

[30] Beaman BL, Maslan S 1977 Effect of cyclophosphamide on experimental Nocardia asteroides infection in mice. Infect Immun 16:995–1004. 330400 10.1128/iai.16.3.995-1004.1977 PMC421062

[31] Levenson VJ, Mallett CP, Hale TL 1995 Protection against local Shigella sonnei infection in mice by parenteral immunization with a nucleoprotein subcellular vaccine. Infect Immun 63:2762–2765. 7790095 10.1128/iai.63.7.2762-2765.1995 PMC173369

[32] Haggerty RM, John DT 1978 Innate resistance of mice to experimental infection with Naegleria fowleri. Infect Immun 20:73–77. 669800 10.1128/iai.20.1.73-77.1978 PMC421553

[33] Molnàr C, Hevessy Z, Rozgonyi F, Gemmell CG 1994 Pathogenicity and virulence of coagulase negative staphylococci in relation to adherence, hydrophobicity, and toxin production in vitro. J Clin Pathol 47:743–748. 7962630 10.1136/jcp.47.8.743 PMC502150

[34] Finke M, Duchêne M, Eckhardt A, Domdey H, von Specht BU 1990 Protection against experimental Pseudomonas aeruginosa infection by recombinant P. aeruginosa lipoprotein I expressed in Escherichia coli. Infect Immun 58:2241–2244. 2114360 10.1128/iai.58.7.2241-2244.1990 PMC258803

[35] Yoshida S, Mizuguchi Y 1986 Multiplication of Legionella pneumophila Philadelphia-1 in cultured peritoneal macrophages and its correlation to susceptibility of animals. Can J Microbiol 32:438–442. 3719461 10.1139/m86-083

[36] Smith IM, Wilson AP, Hazard EC, Hummer WK, Dewey ME 1960 Death from staphylococci in mice. Infect Dis 103:369–378.

[37] Mojgani N, Maldjae V, Rahbar M, Mirafzali SM, Khoshnood S, Hatami A 2013 Intraperitoneal inoculation of Haemophilus influenzae local isolates in BALB/c mice model in the presence and absence of virulence enhancement agents. Indian J Med Microbiol 31:148–153. doi:10.4103/0255-0857.115236. 23867671

[38] Singh KV, Qin X, Weinstock GM, Murray BE 1998 Generation and testing of mutants of Enterococcus faecalis in a mouse peritonitis model. J Infect Dis 178:1416–1420. 9780263 10.1086/314453

[39] Cheers C, McKenzie IF 1978 Resistance and susceptibility of mice to bacterial infection: genetics of listeriosis. Infect Immun 19:755–762. 305895 10.1128/iai.19.3.755-762.1978 PMC422253

[40] Welkos SL, Keener TJ, Gibbs PH 1986 Differences in susceptibility of inbred mice to Bacillus anthracis. Infect Immun 51:795–800. 3081444 10.1128/iai.51.3.795-800.1986 PMC260968

[41] Parker JC, Whiteman MD, Richter CB 1978 Susceptibility of inbred and outbred mouse strains to Sendai virus and prevalence of infection in laboratory rodents. Infect Immun 19:123–130. 203530 10.1128/iai.19.1.123-130.1978 PMC414057

